# European registry of type A aortic dissection (ERTAAD) - rationale, design and definition criteria

**DOI:** 10.1186/s13019-021-01536-5

**Published:** 2021-06-10

**Authors:** Fausto Biancari, Giovanni Mariscalco, Hakeem Yusuff, Geoffrey Tsang, Suvitesh Luthra, Francesco Onorati, Alessandra Francica, Cecilia Rossetti, Andrea Perrotti, Sidney Chocron, Antonio Fiore, Thierry Folliguet, Matteo Pettinari, Angelo M. Dell’Aquila, Till Demal, Lenard Conradi, Christian Detter, Marek Pol, Peter Ivak, Filip Schlosser, Stefano Forlani, Govind Chetty, Amer Harky, Manoj Kuduvalli, Mark Field, Igor Vendramin, Ugolino Livi, Mauro Rinaldi, Luisa Ferrante, Christian Etz, Thilo Noack, Stefano Mastrobuoni, Laurent De Kerchove, Mikko Jormalainen, Steven Laga, Bart Meuris, Marc Schepens, Zein El Dean, Antti Vento, Peter Raivio, Michael Borger, Tatu Juvonen

**Affiliations:** 1 Heart and Lung Center, Helsinki University Hospital, and University of Helsinki, P.O. Box 340, Haartmaninkatu 4, 00029 Helsinki, Finland; 2 Research Unit of Surgery, Anesthesia and Critical Care, University of Oulu, Oulu, Finland; 3 Department of Cardiac Surgery, Glenfield Hospital, University Hospitals of Leicester, Leicester, UK; 4 Southampton University Hospital, Southampton, UK; 5 UK Aortic Surgery Group, Wessex Cardiothoracic Centre, Division of Cardiac Surgery, Southampton University Hospital, Southampton, UK; 6 Division of Cardiac Surgery, University of Verona Medical School, Verona, Italy; 7 Department of Cardio-Thoracic Surgery, Jean Minjoz University Hospital, Besançon, France; 8 Service de Chirurgie Thoracique et Cardio-vasculaire, Hôpital Henri Mondor, Assistance Publique - Hôpitaux de Paris, Créteil, France; 9 Department of Cardiovascular Surgery, Ziekenhuis Oost-Limburg, Genk, Belgium; 10 Department of Cardiothoracic Surgery, Münster University Hospital, Münster, Germany; 11 Department of Cardiovascular Surgery, German Aortic Centre Hamburg, University Heart & Vascular Centre Hamburg, Hamburg, Germany; 12 Institute of Clinical and Experimental Medicine, Prague, Czech Republic; 13 Northern General Hospital, Herries Road, Sheffield, UK; 14 Liverpool Cardiovascular Surgery, Liverpool Heart and Chest Hospital, Faculty of Health and Life Sciences, Liverpool Centre for Cardiovascular Science, University of Liverpool, Liverpool, UK; 15 Cardiac Surgery Department, University of Udine, Udine, Italy; 16 Department of Cardiac Surgery, University of Turin, Turin, Italy; 17 Leipzig Heart center, Leipzig, Germany; 18 Cardiovascular and Thoracic Surgery, Saint-Luc’s Hospital, Catholic University of Louvain, Brussels, Belgium; 19 Department of Cardiac Surgery, University Hospital Antwerp, Edegem, Belgium; 20 Cardiac Surgery, University Hospitals Leuven, Leuven, Belgium; 21 Department of Cardiac Surgery, AZ St-Jan, Bruges, Belgium

**Keywords:** Aortic dissection, Stanford type A, Ascending aorta, Aortic arch, Emergency

## Abstract

**Background:**

Acute Stanford type A aortic dissection (TAAD) is a life-threatening condition. Surgery is usually performed as a salvage procedure and is associated with significant postoperative early mortality and morbidity. Understanding the patient’s conditions and treatment strategies which are associated with these adverse events is essential for an appropriate management of acute TAAD.

**Methods:**

Nineteen centers of cardiac surgery from seven European countries have collaborated to create a multicentre observational registry (ERTAAD), which will enroll consecutive patients who underwent surgery for acute TAAD from January 2005 to March 2021. Analysis of the impact of patient’s comorbidities, conditions at referral, surgical strategies and perioperative treatment on the early and late adverse events will be performed. The investigators have developed a classification of the urgency of the procedure based on the severity of preoperative hemodynamic conditions and malperfusion secondary to acute TAAD. The primary clinical outcomes will be in-hospital mortality, late mortality and reoperations on the aorta. Secondary outcomes will be stroke, acute kidney injury, surgical site infection, reoperation for bleeding, blood transfusion and length of stay in the intensive care unit.

**Discussion:**

The analysis of this multicentre registry will allow conclusive results on the prognostic importance of critical preoperative conditions and the value of different treatment strategies to reduce the risk of early adverse events after surgery for acute TAAD. This registry is expected to provide insights into the long-term durability of different strategies of surgical repair for TAAD.

**Trial registration:**

ClinicalTrials.gov Identifier: NCT04831073.

## Background

Acute Stanford type A aortic dissection (TAAD) is a life-threatening condition. Surgery is usually performed as a salvage procedure and is associated with increased postoperative early mortality and morbidity [1]. Although early mortality has declined during the last years [2], the recent The Nordic Consortium for Acute Type A Aortic Dissection registry including 1189 patients operated from 2005 to 2015 in 8 centers showed that 30-day mortality after surgery for acute TAAD was as high as 18% [3]. The multicentre, prospective German Registry for Acute Aortic Dissection type A including 2137 TAAD patients operated from 2006 and 2010 documented a 30-day mortality of 16.9% [4]. A more recent analysis of the Society of Thoracic Surgeon database including 7353 patients operated from 2014 and 2017 for acute TAAD reported a 30-day mortality of 17% [5]. Furthermore, surgery for TAAD is often complicated by major adverse events such as stroke [5] and acute kidney failure [6], which may have a significant impact on late survival. In this scenario of significant postoperative mortality and morbidity, surgeon faces the controversial issue of the extent of surgical repair for acute TAAD by avoiding a major surgical repair, which may increase the risk of early adverse events, and by resecting segments of the aorta which otherwise may expose the patient to the risk of late aortic-related complications [7, 8]. Understanding the balance between the patient’s conditions which may not allow extensive procedure and those treatment strategies which may limit the risk of late adverse events in patients who remain alive long after the surgery is essential for an appropriate management of TAAD. However, previous registries [1, 3] did not provide information on the long-term durability of these procedures. We have planned the multicenter European registry of surgery for acute TAAD (ERTAAD) to evaluate the contemporary early outcomes and the durability of different surgical strategies for acute TAAD at 15 years in a large study population.

## Methods

The ERTAAD is an observational registry including patients who underwent surgery for acute TAAD at 19 centers of cardiac surgery located in seven European countries (Belgium, Czech Republic, Finland, France, Germany, Italy and the United Kingdom) (Table 1) from January 1, 2005 to March 31, 2021. Data will be retrospectively collected on patients treated during the study period and the aim is to gather further data for future clinical research on this topic. Data on consecutive patients with acute TAAD will be collected into a Microsoft Access datasheet (Redmond, Washington, USA) with pre-specified baseline, operative and outcome variables. Permission to conduct this study will be requested from institutional or national review boards according to local legislation.

**Table 1:** Participating centers

Participating centers	
1. Ziekenhuis Oost-Limburg, Genk, Belgium	
2. Saint-Luc’s Hospital, Brussels, Belgium	
3. Sint-Jan Hospital, Bruges, Belgium	
4. Antwerp University Hospital, Edegem, Belgium	
5. University Hospitals Leuven, Leuven, Belgium	
6. Institute of Clinical and Experimental Medicine, Prague, Czech Republic	
7. Helsinki University Hospital, Helsinki, Finland	
8. Jean Minjoz University Hospital, Besançon, France	
9. Hôpital Henri Mondor, Assistance Publique - Hôpitaux de Paris, Créteil, France	
10. Leipzig Heart Center, Leipzig, Germany	
11. University Heart and Vascular Centre Hamburg, Hamburg, Germany	
12. Münster University Hospital, Münster, Germany	
13. University of Turin, Turin, Italy	
14. University of Verona Medical School, Verona, Italy	
15. University of Udine, Udine, Italy	
16. Glenfield Hospital, University Hospitals of Leicester, Leicester, UK	
17. Liverpool Heart and Chest Hospital, Liverpool, UK	
18. Sheffield Teaching Hospital, Sheffield, UK	
19. Southampton University Hospital, Southampton, UK	

### Inclusion criteria

- TAAD or intramural hematoma involving the ascending aorta;
- Patients aged > 18 years:
- Symptoms started within 7 days from surgery;
- Primary surgical repair of acute TAAD;
- Any other major cardiac surgical procedure concomitant with surgery for TAAD.

### Exclusion criteria

- Patients aged < 18 years;
- Onset of symptoms > 7 days from surgery;
- Prior procedure for TAAD;
- Retrograde TAAD (with primary tear located in the descending aorta);
- Concomitant endocarditis;
- TAAD secondary to blunt or penetrating chest trauma.

## Clinical and operative variables and their definition criteria

### Individual surgeon’s experience

Surgeon having performed performing at least 20 elective or urgent procedures on the ascending aorta/aortic arch in the preceding year.

### Baseline and postoperative laboratory parameters

Baseline levels of hemoglobin, creatinine, platelets, arterial lactate, cardiac enzymes and C-reactive protein as well as postoperative lowest levels of hemoglobin and platelets and postoperative peak levels of creatinine and arterial lactate will be collected.

### Preoperative antithrombotic drugs

Exposure to any of the following antithrombotic drugs within 2 days before surgery: aspirin, clopidogrel, ticagrelor, prasugrel, ticlopidine, low-molecular weight heparin, unfractionated heparin, fondaparinux, direct oral anticoagulants and/or warfarin.

### Renal function

Renal function will be classified according to the estimated glomerular filtration rate (eGFR) calculated using the Chronic Kidney Disease Epidemiology Collaboration (CKD-EPI) equation [9] and the European Kidney Function Consortium (EKFC) equation [10]. The severity of renal failure will be classified in different stages as listed in Table 2.

**Table 2:** Stages of chronic kidney disease

Stages	eGFR (mL/min/1.73 m^**2**^)
1	≥90
2	60–89
3a	45–59
3b	30–44
4	15–29
5	< 15 or dialysis

*eGFR* estimated glomerular filtration rate

### Genetic syndromes

Genetic syndromes may lead to aortopathies and TAAD [11]. Information on any specific genetic syndrome associated with TAAD will be collected in this registry.

### Arterial hypertension

Systemic arterial pressure > 150/80 mmHg or use of antihypertensive drugs.

### Diabetes

Hyperglycemia requiring treatment with insulin or oral drugs.

### Prior stroke

Any preoperative focal or global neurological syndrome caused by ischemia or hemorrhage not resolving within 24 h. It refers to a neurological event occurring any time before hospitalization for TAAD, but not to a neurological event related to acute TAAD.

### Prior transient ischemic attack

Any preoperative focal or global neurological syndrome caused by ischemia or hemorrhage resolving within 24 h. It refers to a neurological event occurring any time before hospitalization for TAAD, but not to a neurological event related to acute TAAD.

### Pulmonary disease

Use of bronchodilators and/or steroids for lung diseases [12].

### Extracardiac arteriopathy

One or more of the following: lower limb claudication, critical limb ischemia, carotid occlusion or > 50% stenosis, major amputation for arterial disease, previous or planned intervention on the abdominal aorta or on the extremities or carotid arteries [12].

### Poor mobility

Severe impairment of mobility due to musculoskeletal or neurological dysfunction [12].

### Moderate-to-severe frailty

Patient needs assistance because of moderate-to-severe physical and cognitive impairment (score > 5 on the Rockwood Clinical Frailty Scale) [13].

### New York Heart Association functional classes

Functional classes according to the New York Heart Association classification whose definition criteria are summarized in Table 3 [14].

**Table 3:** New York Heart Association functional classes

Class	Patient symptoms
I	No limitation of physical activity. Ordinary physical activity does not cause undue fatigue, palpitation, dyspnea, or anginal pain.
II	Slight limitation of physical activity. Comfortable at rest. Ordinary physical activity results in fatigue, palpitation, dyspnea or anginal pain.
III	Marked limitation of physical activity. Comfortable at rest. Less than ordinary activity causes fatigue, palpitation, dyspnea or anginal pain.
IV	Inability to carry on any physical activity without discomfort. Symptoms of heart failure or the anginal syndrome may be present even at rest. If any physical activity is undertaken, discomfort is increased.

### Recent myocardial infarction

Myocardial infarction within 90 days from the index surgery [12]. It includes acute coronary malperfusion (i.e. any changes in ST level in electrocardiogram and/or an increase in cardiac enzymes).

### Pulmonary hypertension

Systolic pulmonary artery pressure on arrival in the operating room classified in three categories: < 31 mmHg, 31–55 mmHg and > 55 mmHg [12].

### Critical preoperative state

Ventricular tachycardia or ventricular fibrillation or aborted sudden death, preoperative cardiac massage, preoperative ventilation before anesthetic room, preoperative inotropes, preoperative acute renal failure (anuria or oliguria < 10 ml/hr) [12]. These conditions will be reported as separate variables.

### Urgency of the procedure

Urgency of the procedure will be classified in five categories based on increasing severity of hemodynamic instability and the need and timing of cardiopulmonary resuscitation (Table 4).

**Table 4:** Urgency of the procedure.*

Urgency	Definition
Urgent	Scheduled procedure performed in paucisymptomatic patients with stable hemodynamic conditions during the index hospitalization since the next working day from admission
Emergency grade 1	Procedure performed in patients with stable conditions and without malperfusion before the beginning of the next working day
Emergency grade 2	Procedure performed in patients with hemodynamic instability despite the use of inotropes and/or any malperfusion before the beginning of the next working day - no cardiopulmonary resuscitation with chest compression required
Salvage grade 1	Procedure performed in patients requiring cardiopulmonary resuscitation with external chest compressions and/or open chest cardiac massage between induction of anesthesia and initiation of cardiopulmonary bypass
Salvage grade 2	Procedure performed in patients requiring cardiopulmonary resuscitation with external chest compressions en route to the operating theatre or before induction of anesthesia

Further details on the definition criteria of the urgency of the procedure are reported in the text

Urgent procedure refers to a procedure which was scheduled for the next working day from admission or later during the index hospitalization. These patients were hemodynamically stable and paucisymptomatic. In these patients, TAAD was considered not to cause to malperfusion and/or aortic rupture within the next few hours/days.

Emergency grade 1 refers to any procedure which was performed before the beginning of the next working day in symptomatic or paucisymptomatic patients with stable hemodynamic conditions and without signs of malperfusion. The procedure was performed within a few hours after admission to the hospital.

Emergency grade 2 refers to any procedure which was performed before the beginning of the next working day because of hemodynamic instability despite the use of inotropes and/or of malperfusion. These patients did not require cardiopulmonary resuscitation. The procedure was performed immediately after admission to the hospital or worsening of the patients’ conditions.

Salvage grade 1 refers to any procedure which was performed in patients requiring cardiopulmonary resuscitation with external chest compressions and/or open cardiac massage between induction of anesthesia and initiation of cardiopulmonary bypass. The procedure was performed when the conditions of the patients worsened after induction anesthesia due to acute heart failure, cardiac tamponade and/or frank rupture of the aorta. These patients required either external chest compressions before sternotomy and/or cardiac massage after sternotomy and prompt initiation of cardiopulmonary bypass.

Salvage grade 2 refers to any procedure which was performed in patients requiring cardiopulmonary resuscitation with external chest compressions en route to the operating theatre or prior to induction of anesthesia. These patients might have required also cardiac massage after sternotomy and prompt initiation of cardiopulmonary bypass. In these patients, the operation was performed on compassionate basis without knowledge of frank rupture of the aorta or severe end-organ injury.

### Neurological status immediately before procedure

Unconsciousness before sedation, hemiplegia/hemiparesis, paraplegia/paraparesis, dysarthria/aphasia, vision loss, confusion, or intubated/sedated at arrival. These conditions will be reported as separate variables.

### Malperfusion

Malperfusion refers to acute organ ischemia secondary to aortic branch vessel hypoperfusion. This severe condition is usually classified based on clinical signs and symptoms [15, 16]. Herein, myocardial malperfusion will be defined as any changes in ST level in electrocardiogram and/or an increase in cardiac enzymes. Cerebral malperfusion will be defined as acute preoperative stroke. Spinal malperfusion will be defined as acute paraparesis/paraplegia. Mesenteric malperfusion will be as sudden, mild-to-severe abdominal pain with or without nausea and vomiting, which is accompanied or not by rectal bleeding or bloody diarrhea [17]. Renal malperfusion will be defined as anuria/oliguria. Peripheral malperfusion will be defined as loss of pulse with or without sensory or motor deficits of any limb.

### Preoperative computed tomography findings

Preoperative computed tomography scans will be reviewed for evaluation of the extent of aortic dissection/intramural hematoma in different segments of the aorta and its branches. Dissection of the aortic branches will be defined as any intimal flap at the origin of the artery causing stenosis of any severity. This applies also to aortic branches perfused through the false lumen.

Data on maximal diameter of the aortic root, ascending aorta, aortic arch, descending aorta and abdominal aorta will be collected.

### Intramural hematoma

A contained aortic wall hematoma within the media, but without intimal flap formation of the ascending aorta. Patients will be considered as having aortic intramural hematoma when no concomitant aortic intimal flap was observed at preoperative aortic imaging. When intramural hematoma was associated with an intimal flap of any other segment of the aorta, this will be classified as a true aortic dissection.

### Heart valve status and left ventricular function

Data from preoperative echocardiographic evaluation of the aortic valve, mitral valve and left ventricular function will be collected.

### Arterial and venous cannulation sites

It refers to the primary cannulation sites. Any switch to other cannulation site before initiation of cardioplegia will be described.

### Intraoperative findings

Data on the intraoperative findings of the pericardium, ascending aorta and aortic arch will be collected. The extent of aortic dissection at the level of the Valsalva sinuses and morphology of the aortic valve will be described.

### Surgical repair

Data on the type of aortic root/ascending aorta and aortic arch surgical repair will be collected along with data on any concomitant major cardiac surgery procedure. Details regarding the level of aortic anastomosis will be documented. In particular, data on aortic anastomosis suture techniques (i.e. the use and number of layers of Teflon or pericardial patch to reinforce the anastomosis and the aortic wall) and the use of biological glue to the dissected tissues will be retrieved. Double layer reinforcement patch technique refers to an anastomosis suture with a combination of one patch outside the aorta and the other between the dissected tissues or from inside the aorta. Triple layer reinforcement patch technique refers to an anastomosis suture with a combination of one patch outside the aorta, one within the dissected tissues and one from inside the aorta.

### Cardiopulmonary bypass parameters

Data on duration of myocardial ischemia, cardiopulmonary bypass, hypothermic circulatory arrest and retrograde or antegrade cerebral perfusion will be collected. When multiple periods of perfusion or organ ischemia have occurred, the total length of these periods will be reported. The lowest temperature during hypothermic circulatory arrest will be documented.

## Outcomes and their definition criteria

### Hospital death

All-cause death having occurred during the index hospitalization, i.e. at the institution where surgery for TAAD was performed.

### Acute heart failure

Postoperative heart failure requiring prolonged use of inotropes (> 24 h) and/or the insertion of any mechanical circulatory support device.

### Stroke

Acute episode of a focal or global neurological deficit with at least one of the following: change in the level of consciousness, hemiplegia, hemiparesis, numbness, or sensory loss affecting one side of the body, dysphasia or aphasia, hemianopia, amaurosis fugax, or other neurological signs or symptoms consistent with stroke duration of a focal or global neurological deficit ≥24 h; OR < 24 h if available neuroimaging documents a new brain hemorrhage or infarct; OR the neurological deficit results in death [18]. Stroke will be classified as ischemic, hemorrhagic or both.

### Global brain ischemia

Diffuse hypoxic damage as diagnosed at brain imaging and electroencephalography.

### Paraplegia/paraparesis

Bilateral weakness and/or multimodality sensory disturbance below the level of the ischemic spinal lesion.

### Mesenteric ischemia

Abdominal pain with or without nausea and vomiting and rectal bleeding or bloody diarrhea [17]. Diagnosis is done at imaging, endoscopy and/or surgery.

### Acute kidney injury

It will be defined according to postoperative change in serum creatinine levels and its severity will be stratified according to the Kidney Disease: Improving Global Outcomes (KDIGO) criteria [19] (Table 5). However, in view of the prolonged hospital treatment of TAAD patients, changes in serum creatinine level will be evaluated as occurring during the entire index hospitalization. We recognize that acute kidney injury can also be diagnosed according to urine output measure. However, this method is not feasible in retrospectively gathered data and therefore it will not be considered in the present study. Dialysis will be defined as temporary when discontinued and permanent if continued at the time of discharge from the institution where surgery for acute TAAD was performed.

**Table 5:** Definition criteria of postoperative acute kidney injury.^a^

Stages	Serum creatinine
1	1.5–1.9 times baseline OR ≥0.3 mg/dL (≥26.5 micromol/L) increase
2	2–2.9 times baseline
3	≥3.0 times baseline OR Increase to ≥4 mg/dL (≥353.6 micromol/L) OR Initiation of renal replacement therapy

^a^Changes in serum creatinine level occurring during the index hospitalization

### Perioperative bleeding

Data on number of transfused units of red blood cell will be collected. The E-CABG bleeding classification has been proposed as a simple classification of perioperative bleeding [20] and it has been shown being comparable to the Universal Definition of Perioperative Bleeding [21] in predicting early mortality [22]. Herein we will adopt a simplified version of the E-CABG perioperative classification. This classification defines severe bleeding as transfusion of more than 4 units of red blood cells during and after surgery and/or reoperation for excessive intrathoracic bleeding (Table 6).

**Table 6:** Simplified E-CABG perioperative bleeding classification [20]

Grades	Intervention for treatment of bleeding
0	No RBC transfusion or transfusion of 1 unit of RBCs
1	Transfusion of 2–4 units of RBCs
2	Transfusion of 5–10 units of RBCs and/or reoperation for intrathoracic bleeding
3	Transfusion of > 10 units of RBCs

*RBC* red blood cell

### Reoperation for bleeding

Chest reopening for excessive bleeding. It qualifies as a reoperation for bleeding also any reoperation for excessive bleeding in patients in whom the sternum was left open. Chest reopening for hemodynamic instability without excessive bleeding as well as pericardial/pleural puncture or chest drain insertion for retained blood syndrome do not qualify as reoperations for bleeding.

### Deep sternal wound infection/mediastinitis

Proven infection involving deep sternal wound tissues and/or mediastinum.

### Procedures for vascular complications

Any vascular and endovascular procedure for ischemic or bleeding complications.

These complications will encompass neurologic complications, mesenteric ischemia, upper and lower limb ischemia as well as bleeding from the aorta and its branches.

### Mechanical circulatory support

The use of intra-aortic balloon pump and/or venoarterial extracorporeal membrane oxygenation for postoperative acute heart failure. The duration of treatment of these salvage therapies will be documented.

### Length of stay in the intensive care unit

Overall length of treatment in the intensive care unit, including readmissions, at the institution where surgery for TAAD was performed.

### Late outcomes

Data on patient’s survival status will be collected. Patients lost to follow-up will be reported.

Data on any repeat surgical or endovascular procedure on the aorta will be reported along with its urgency, indications and aortic segment treated.

## Sample size calculation

The impact of total aortic arch repair compared to more limited aortic resection on the early and late outcomes will be one of the main topics of research from this registry. Therefore, in the light of the expected limited frequency of this surgical strategy, we performed a sample size analysis considering the extent of distal aortic resection. Despite the limitation of a pooled analysis which did not take in to account the cumulative incidences from competing risk analysis and the segment of repeat operation, Poon et al. [23] estimated a pooled hazard ratio on 0.73 (95%CI 0.56–1.18) of freedom from any late aortic reoperation for total arch replacement. Based on this hazard ratio, a sample size of 163 patients per study groups would be enough to reject the null hypothesis (alpha 0.05, power 0.80). Pan et al. [24] reported a freedom from distal reoperation after ascending aortic resection at 8 years of 95.3% which was not statistically different from that of hemiarch or total aortic arch repair (92.4%, *p* = 0.22). For the sample size calculation of a non-inferiority trial, we assumed a freedom rate from distal aortic reoperation of 95% and we considered that a difference in the reoperation rate as large as 10% in favor of a total aortic arch repair still allow the less extensive repair to be non-inferior (delta = 0.1, power 0.90, one-sided confidence 97.5%), the required sample size would be 100 patients per each group. We assume that about 5% of patients have undergone total arch repair in this collaborative registry and therefore a study population of 4000 patients would be enough to investigate the impact of total aortic arch repair in preventing late aortic reoperation in patients with acute TAAD. The participating hospitals are expected to provide a sample size of more than 6000 patients (i.e. at least a mean of > 20 procedures for acute TAAD each year), which would prevent a type II error.

## Statistical analysis

Statistical analyses will be conducted using Stata v. 15.1 (StataCorp LLC, Texas, USA) and SPSS v. 27.0 (IBM Corporation, Armonk, NY, USA) statistical softwares. Continuous variables and outcomes will be reported as mean and standard deviation as well as median and interquartile range. Categorical variables and outcomes will be reported as counts and percentages. A *p* < 0.05 will be set for statistical significance. Differences between the study groups will be evaluated using the Fisher’s exact test, the Chi-square test, the linear-by-linear association test, the Mann-Whitney test and the Kruskall-Wallis test. The McNemar test and paired samples T-test will be used in the paired groups. Propensity score matching will be performed to balance treatment strategies for baseline differences. A propensity score will be estimated using a non-parsimonious multilevel mixed-effects logistic regression considering the cluster effect of the participating centers whose results may be affected by the referral pathway, surgical techniques and perioperative care. Multilevel mixed-effects logistic regression model will include all available baseline variables. The estimated propensity score will be employed for inverse propensity score weighting analysis and one-to-one propensity score matching analysis using a caliper width of 0.1 to 0.2 of the standard deviation of the estimated logit. Standardized differences < 0.10 will be considered an acceptable imbalance between the matched cohorts.

Survival analysis will be performed using the Kaplan-Meier method with the log-rank test and the Cox proportional hazards method. Competing risk analysis with the Gray’s test will be performed for late non-fatal adverse events because patient’s death might hinder the observation of these events. Regression models will include covariates with *p* < 0.05 or *p* < 0.2 in univariate analysis according to the number of covariates of interest. Risk estimates will reported as hazard ratios and subdistribution hazard ratios with their 95% confidence intervals.

Logistic and linear regression analyses will be employed to identify independent risk factors for early adverse events with and without a stepwise approach. For the purpose of developing a risk scoring method from this registry, the study cohort will be randomly divided into two datasets, i.e. the derivation data set (75.0% of patients) and the validation data set (25.0% of patients). Univariate analysis will be performed on all candidate covariates of the derivation dataset to identify predictors of in-hospital mortality. Multiple logistic regression analysis will be then performed using the derivation dataset. A stepwise procedure with a bootstrap approach will be used, and 100 samples will be extracted with a size of 70% of the derivation dataset. A stepwise procedure will be applied to each sample (probability to stay = 0.05; probability to entry = 0.1). Variables selected in at least 50% of the stepwise procedures will be included in the regression model. Additional covariates were selected using a forward selection comparing the Akaike information criterion for the models with and without each covariate. The model with the lowest Akaike information criterion will be selected for each forward step, until the inclusion of a new covariate determined an increase in the Akaike information criterion value. The model will be tested in the validation data set for calibration, using the Hosmer–Lemeshow test, and for discrimination using the receiver-operating characteristic curve analysis. A comparison of the predictive ability of the new score with the current risk scoring methods for prediction of in-hospital mortality will be performed in the overall dataset as well as in the validation dataset using the DeLong test. The improvement of discrimination of the new score will be estimated by calculating the net reclassification index and the integrated discrimination improvement. Finally, to evaluate the adaptability of the new score for prediction of late mortality, Kaplan–Meier curves stratified by deciles of the new score will be plotted.

Interinstitutional and between-surgeons differences in terms of early outcomes will be evaluated using mixed-effects logistic regression. The risk-adjusted rate of adverse binary events at each center will estimated by dividing the observed number of adverse events by the expected number of adverse events, and by multiplying this ratio by the average event rate of the overall series. The expected numbers of adverse events will be calculated with logistic regression. The estimated risk-adjusted rates of adverse events will be summarized as odds ratios and their 95% confidence intervals in caterpillar plots with the x-axis ordered for increasing adjusted rates.

## Discussion

### Implications for the treatment of TAAD patients

Analysis of the data from this collaborative registry will allow analysis of contemporary results of a large number of TAAD patients with a rather long follow-up period. We expect that the multicentre nature of this registry will allow reduction in the risk of bias related to institutional volume and surgical experience. In fact, all the participating centers have an annually rate of at least 25 procedures for acute TAAD and have an aortic surgery program which would facilitate an adequate follow-up and management of possible late aortic events after primary repair of TAAD. Therefore, we expect to gather data which will provide important insights into the assessment of the operative risk of patients with acute TAAD and conclusive results on the value of different surgical and perfusion strategies for this emergency condition.

### Limitations

The retrospective nature is the main limitation of this registry. However, our aim is to investigate the outcome at 15 years of these patients, and this may not be feasible with a prospective registry. Second, the prognostic impact of different treatment strategies will be analyzed using several statistical methods to adjust for baseline differences. However, the lack of randomization does not prevent the effects of confounding variables. Third, perioperative care may significantly vary between institutions as well as individual surgeons and their effects should be considered in all analyses.

### Strengths

This registry will include detailed data on patients’ characteristics, operative strategies and outcomes from centers with large volume of cardiac surgery and significant experience in the treatment of acute and chronic aortic diseases. This may reduce the risk of bias related to limited experience in the surgical treatment of aortic diseases. This multicenter registry will allow recruiting of more than 6000 patients, which is expected to decrease the risk of type II errors. The long follow-up of this study will allow reliable analysis of the durability of different surgical repair strategies.

## Conclusions

The ERTAAD is expected to recruit a large number of patients who underwent surgery for acute TAAD. This multicenter study will allow the identification of risk factors associated with major adverse events and of strategies for adequate end-organ protection. The rather long follow-up of this registry is expected to provide solid data on the durability of different types of aortic repair strategies.

## Data Availability

This article is not based on individual patient data.

## Abbreviations

CKD-EPI: Chronic Kidney Disease Epidemiology Collaboration
E-CABG: European Coronary Artery Bypass Grafting registry
EKFC: European Kidney Function Consortium
ERTAAD: European Registry of Type A Aortic Dissection
KDIGO: Kidney Disease: Improving Global Outcomes
RBC: Red Blood Cell
TAAD: Type A Aortic Dissection
