# Correction to: European registry of type A aortic dissection (ERTAAD) - rationale, design and definition criteria

**DOI:** 10.1186/s13019-021-01606-8

**Published:** 2021-08-09

**Authors:** Fausto Biancari, Giovanni Mariscalco, Hakeem Yusuff, Geoffrey Tsang, Suvitesh Luthra, Francesco Onorati, Alessandra Francica, Cecilia Rossetti, Andrea Perrotti, Sidney Chocron, Antonio Fiore, Thierry Folliguet, Matteo Pettinari, Angelo M. Dell’Aquila, Till Demal, Lenard Conradi, Christian Detter, Marek Pol, Peter Ivak, Filip Schlosser, Stefano Forlani, Govind Chetty, Amer Harky, Manoj Kuduvalli, Mark Field, Igor Vendramin, Ugolino Livi, Mauro Rinaldi, Luisa Ferrante, Christian Etz, Thilo Noack, Stefano Mastrobuoni, Laurent De Kerchove, Mikko Jormalainen, Steven Laga, Bart Meuris, Marc Schepens, Zein El Dean, Antti Vento, Peter Raivio, Michael Borger, Tatu Juvonen

**Affiliations:** 1 Heart and Lung Center, Helsinki University Hospital, and University of Helsinki, Haartmaninkatu 4, P.O. Box 340, 00029 Helsinki, Finland; 2 Anesthesia and Critical Care, Research Unit of Surgery, University of Oulu, Oulu, Finland; 3 Department of Cardiac Surgery, Glenfield Hospital, University Hospitals of Leicester, Leicester, UK; 4 Southampton University Hospital, Southampton, UK; 5 UK Aortic Surgery Group, Wessex Cardiothoracic Centre, Division of Cardiac Surgery, Southampton University Hospital, Southampton, UK; 6 Division of Cardiac Surgery, University of Verona Medical School, Verona, Italy; 7 Department of Cardio-Thoracic Surgery, Jean Minjoz University Hospital, Besançon, France; 8 Service de Chirurgie Thoracique et Cardio-vasculaire, Hôpital Henri Mondor, Assistance Publique - Hôpitaux de Paris, Créteil, France; 9 Department of Cardiovascular Surgery, Ziekenhuis Oost-Limburg, Genk, Belgium; 10 Department of Cardiothoracic Surgery, Münster University Hospital, Münster, Germany; 11 Department of Cardiovascular Surgery, German Aortic Centre Hamburg, University Heart and Vascular Centre Hamburg, Hamburg, Germany; 12 Institute of Clinical and Experimental Medicine, Prague, Czech Republic; 13 Northern General Hospital, Herries Road, Sheffield, UK; 14 Liverpool Cardiovascular Surgery, Liverpool Heart and Chest Hospital, Faculty of Health and Life Sciences, Liverpool Centre for Cardiovascular Science, University of Liverpool, Liverpool, UK; 15 Cardiac Surgery Department, University of Udine, Udine, Italy; 16 Department of Cardiac Surgery, University of Turin, Turin, Italy; 17 Leipzig Heart Center, Leipzig, Germany; 18 Cardiovascular and Thoracic Surgery, Saint-Luc’s Hospital, Catholic University of Louvain, Brussels, Belgium; 19 Department of Cardiac Surgery, University Hospital Antwerp, Edegem, Belgium; 20 Cardiac Surgery, University Hospitals Leuven, Leuven, Belgium; 21 Department of Cardiac Surgery, AZ St-Jan, Bruges, Belgium

## Correction to: Journal of Cardiothoracic Surgery (2021) 16:171 https://doi.org/10.1186/s13019-021-01536-5

The original article [1] erroneously contained copyediting comments in the Methods section which have since been removed.
